# Progress in Surgery

**Published:** 1898-01-15

**Authors:** 


### PROGRESS IN SURGERY.

### SURGERY OF THE PERITONEUM, &c.

(*Continued from page 260.*)

**Retro-peritoneal and Perirenal Lipomata** occur most frequently, according to Adam,[14] around the kidneys, but may be met with in the mesentery, radix mesenterii, iliac fossa, or elsewhere. Two cases related by him have a similar history—long duration, slow growth, very little general disturbance, no pain, and extreme emaciation. In both the growth consisted of fatty tissue, in which the kidney was embedded. Both were retroperitoneal in position, and crossed by a length of large intestine. These growths are rare, but are met with more frequently in the female than in the male, in the proportion of 25 to 16. Middle and later life are the periods at which they usually occur. Growth is always slow; generally two to three years elapse between the first recognition and the death of the patient. The majority are more than 20 lb. in weight, and one was 63 lb. In many the definite presence of fluctuation has led to erroneous diagnosis. Intestine usually passes in front of the growth. Eventually œdema of the lower extremities appears, due to venous obstruction in the abdomen. The growth is generally a myxo-lipoma, but other forms of connective tissue are met with as fibro-chondro-osteo-lipomata, or lipo-sarcomata. Of the 42 cases collected, in 26 the growth was wholly or almost wholly removed. In 46·1 per cent. the operation was successful. Generally there are but few adhesions. The peritoneum covering the growth is often smooth and glistening, and the mass usually peels out fairly easily. The chief difficulty lies in the presence of intestine which has been carried forward with the mesenteric vessels. Unless great care be taken, the blood supply of the gut will be interfered with. The growth should, if possible, be approached by a lateral or lumbar incision, and free resection of intestine performed when necessary.

**Abdominal Contusions with Visceral Lesions.**—Gilliam[15] says exploratory incision for the above-named injury is indicated—(1) Where blood is found in the ejecta of the stomach or bowels, or in the urine; (2) where abdominal rigidity, tympanites, or other inferential signs of visceral lesions exist; (3) where patient complains of burning pain, or a sense of grave internal injury; (4) where there is profound, remittent, or recurrent shock; and (5) where from the nature of the injury it is probable that visceral lesion has resulted. The patient in all cases should be watched closely for 48 hours, during which time any untoward manifestation should be the signal for exploration.

**Abdominal Cystic Tumours** in the male are very rare, but Hearn[16] has met with a congenital, multilocular omental cyst in a boy of eight years. Beyond the sense of weight in the abdomen and frequency of micturition, the symptoms were practically nil. The whole cyst, which contained a dark greenish-brown fluid, was dissected out, and the boy made a good recovery. The contents had a specific gravity of 1,009, reaction slightly alkaline, no urea, but a large amount of albumen.

**A Test for Suppuration** is given by Schnitzler,[17] which furnishes an exact and reliable index of the beginning of suppurative processes. In such cases as appendicitis, some definite reliable sign that the catarrhal process has become a suppurative one has long been a desideratum. The test consists in a characteristic iodin reaction in the blood. This is not attributable to the presence of the extra-cellular granules described by Ehrlich, but is an intra-cellular reaction. Ehrlich's iodin gum solution is used, and the extra-cellular granules are found increased in suppurative processes. Besides this, however, some of the polynuclear neutrophile leucocytes exhibit a characteristic yellowish to brownish colour. This does not occur in the eosinophiles, and only in certain polynuclear cells. It was observed but three times in over a thousand preparations in mono-nuclear cells. This intra-cellular iodin reaction occurs only in acute suppuration, and disappears twenty-four hours after an abscess has been evacuated. Its appearance seems to coincide very closely with the occurrence of suppuration, and where an inflammatory exudation does not become purulent, but goes on to resorption, it does not occur. The more rapid the purulent degeneration, and the more invasive it is, the more intense is the intra-cellular reaction to the iodin solution. Where a stationary abscess exists the reaction does not occur, unless the natural resistance is weakened and infective invasion once more begins.

[14] Montreal Med. Journ., Jan. and Feb., 1897; and Med. Chron., July, 1897, p. 268. [15] Columbus Med. Journ., vol. viii., No. 6, 1897; and Amer. Med.-Surg. Bullet., June 25, 1897. [16] Annals of Surg., June, 1897, p. 703. [17] Med. News, Aug. 7, 1897, p. 185.

### SURGERY OF THE ŒSOPHAGUS AND STOMACH.

**The Value of Skiagraphy** in detecting foreign bodies in the œsophagus is discussed by Péan, who states that children can swallow bodies as large as half an inch in diameter, and that the recognition of the presence of obstruction is often delayed because the child swallows liquids without difficulty and does not complain of pain. Skiagraphy not only permits an accurate location of the foreign body in most cases, but determines also the operation of election. After the œsophagus has been exposed, coins, buttons, &c., may be gradually caused to reascend the œsophagus to such a level that they may be extracted from above, thus avoiding the danger and discomfort of opening the gullet.

**Gastrostomy** for stricture of the œsophagus should be performed earlier than is usually done, says Mayo Robson.[2] He prefers a modification of the Ssabanejew-Franks method: A vertical incision, about an inch and a half long, is made over the outer third of the left rectus abdominis, commencing three-quarters of an inch below the costal margin; the fibres of the rectus are separated, and a portion of the stomach is brought out through the incision in the form of a cone, into the base of which four sutures uniting visceral and parietal peritoneum are placed. A transverse incision of half an inch is then made one inch above the upper end of the first cut, and the two incisions are connected by undermining the bridge of skin. The stomach is then passed under this bridge of skin, fixed by two harelip pins in the upper opening, and the lower opening closed. The stomach may be opened at once or later, and the patient fed through a catheter fastened on the end of a funnel. This operation prevents all leakage from the stomach. A similar operation was proposed by Albert, and is advocated by Barling.[3] Senn[4] has performed gastrostomy by a circular valve method as follows: An incision was made 10 cm. long parallel to left costal arch, and part of the stomach wall near the great curvature was raised above the level of the wound in the form of a cone. The base of this cone was constricted by a circular suture involving serous and muscular coats, and the constricted part was supplemented by a collar of omental tissue, which, with the stomach, was then attached to the margins of the wound—except the skin—and the rest of the wound was closed. The projecting part of the stomach was then incised, and a tube passed into the cavity. The flaps were inverted and secured with Lembert sutures, and the tube then removed. The advantages claimed for this method are (1) retention of gastric juice by valve of ventricular origin; (2) closure of fistula during digestion; (3) trifling dislocation of stomach during operation.

**Gastrotomy** was successfully performed by Fricker[5] on a woman 32 years of age, from whom he removed 37 bodies, including a large key, a table fork, two teaspoons, two nails, two hairpins, 12 pieces of glass, a window-fastener, and other objects.

**Cicatricial Stenosis of the Cardiac Orifice of the Stomach** may, according to the experiments of Bozzi,[6] be cured by an operation based on the principle of the Heineke-Mikulicz pyloroplasty. An incision is made from the ensiform cartilage to the eleventh rib. The liver is dislocated to the right by dividing the left lateral ligament, and the stomach dragged downwards and outwards, exposing the œsophageal opening in the diaphragm. The œsophagus is freed by scissors, and the operation field packed with gauze. The gastric contents are "stripped" back, and a clamp applied to the stomach a little below the cardiac orifice. The cicatricial contraction is then dealt with as in pyloroplasty. To be successful, the following conditions are necessary: (1) That the cicatricial stenosis is limited to the cardiac orifice and the adjacent œsophagus, so that a longitudinal incision proportionate to the length of the stenosis can be made; (2) every ulcerative process hindering or preventing cohesion must previously be cured; (3) there should be no adhesions rendering the separation of the œsophagus impossible or too dangerous; (4) the nature of the stricture must be such as to render the performance of the operation justifiable; (5) the general condition of the patient should be fairly good. Whether the first, second, and last conditions are fulfilled can be ascertained by physical examination and measurement of the depth of the stricture, while the third can only be judged to be present from probabilities.

**Gastric Ulcer**, according to Von Leube,[7] rarely calls for surgical treatment, and the author has successfully treated about a thousand cases without operation. The patient should stay in bed, use hot poultices, and drink Carlsbad water. The poultices must be very hot, and must be changed every ten minutes. Pain usually disappears in about five days. Females must not wear corsets, and strict regulation of the diet is indispensable. Of all his cases only 2·4 per cent. died, 1·6 per cent. were not benefited, 21 per cent. showed marked improvement, and 75 per cent. were completely cured. Surgical treatment should be resorted to under the following circumstances: (1) When there are repeated hæmorrhages, causing anæmia. It depends on the nature of the individual case whether an operation should be performed or not where there has been only a simple profuse hæmorrhage, for it may be so abundant that surgical aid comes too late; on the other hand, patients often recover from the acute anæmia caused by a single hæmorrhage. (2) When long-continued medical treatment fails to relieve the pain, gastro-enterostomy may be tried. (3) When there is peri-gastritis caused by adhesion of the stomach to other organs, especially when a sub phrenic abscess has formed. (4) When there is perforation of the ulcer and an opening into the abdominal cavity. The operation must be performed within ten hours after the perforation. The author has only met with two cases of perforation which recovered spontaneously. Mickulicz[8] says that relapses are rather frequent, and most observers' results are not as good as those stated by Von Leube. On the other hand, surgical treatment is now so successful that of the patients operated on only 10 per cent. died. Three methods of operation must be considered: 1. Resection, which is the most dangerous method and not very satisfactory, but must be performed when the case is carcinomatous. 2. Pyloroplasty, which is a more simple method, but can only be carried out when the gastric and duodenal walls are soft. 3. Gastro-enterostomy, which also gives good results, but sometimes the contents of the stomach penetrate into the ascending limb of the jejunum, and Mickulicz therefore recommends that a second anastomosis should be made between the two loops. Operation is indicated: (1) When the patient's life is endangered by hæmorrhage, perforation, and inanition; (2) when prolonged medical treatment has proved useless. Alexander[9] thinks that the correspondence of the pain of gastric ulcer to the ingestion of food must vary greatly according to the position of the ulcer, and quotes a case where an ulcer on the anterior wall and close to the lesser curvature escaped irritation either by food passing over it or being deposited on its floor. Kirkpatrick[10] and Fyffe[11] both report successful operations after perforation. Lauenstein[12] insists on the practical value of preventive plugging with sterilised gauze in cases of operation on the hollow viscera. For example, in the case of a perforating gastric ulcer he would, after exposure of the stomach and irrigation, plug the fistulous opening by a long strip of gauze passed into the stomach. He would then apply the sutures in a loose row, and, on tying these one by one, would gradually remove the gauze. The discharge of any fluid remaining in the stomach during the application of the sutures or the separation of adhesions may thus be avoided. This method is useful also in operations for gangrenous hernia, for cholelithiasis and fistula of the gall-bladder, for cancer of the rectum, and for perforating wound and rupture of the intestine and urinary bladder.

**Gastro-enterostomy,** according to Meyer,[13] is best performed by means of Murphy's anastomosis button: (1) It hastens and simplifies the operation; it enables the patient to be fed through the mouth directly after the operation; the anastomosis is still feasible with its help where proper suturing is impossible; the anastomosis made with it does not contract. (2) In using the button, posterior gastro-enterostomy (von Hacker's operation) is preferable to the anterior one (Wölfler's), because it favours the progress of the button towards the anus. In both methods, however, the button can drop into the stomach. (3) The presence of the button within the stomach has, so far, never done actual harm, and therefore this accident is not to be considered a drawback to its use. (4) In all cases where reduction of time in the operation is of importance the use of the button is indicated and not the suture. (5) There is no reason borne out by practical experience which should prevent us from making use of the advantages of the button in every case of gastro-enterostomy for malignant disease. (6) If the button be used, great emaciation of the patient is no more a contra-indication to this operation than it is to gastrostomy in cancer of the œsophagus. (7) On account of the possible entrance of the button into the stomach, gastro-enterostomy in cases of benign stricture of the pylorus should be done with the help of the suture. Heydenreich[14] divides the accidents which have followed the use of the button into four classes: (1) Where the button did not procure the exact coaptation of the surfaces brought in contact, either because the buttons were too small or were not properly brought together. This can be obviated by using large buttons, and employing Lembert sutures. (2) Where the button prevented the passage of intestinal contents—chiefly in the large intestine. This can be got over by purgatives, but he does not advise its use in the large intestine. (3) Where there was perforation of the intestine, either from defective union or by gangrene, from later pressure by a button of too large calibre. (4) In gastro-enterostomy the button sometimes falls into the stomach. The author does not believe that these accidents should prevent the use of the button.

[1] Annals of Surg., May, 1897. [2] Practitioner, Sept., 1897, p. 242. [3] Birming. Med. Rev., June, 1897, p. 321. [4] Glasgow Med. Journ., July, 1897, p. 77. [5] Internat. Med. Mag., April, 1897, p. 209; and Deutsch. Med. Woch., No. 4, 1897. [6] Annals of Surg., Sept., 1897, p. 391; and Beitr. z. Klin. Chir., Band xviii., Heft 2. [7] Lancet, May 8, 1897, p. 1,305; and Med. Rec., May 29, 1897, p. 789. [8] Ibidem. [9] Brit. Med. Journ., May 29, 1897, p. 1,345. [10] Practitioner, Aug., 1897, p. 157. [11] Austral, Med. Gazette, July 20, 1897, p. 331. [12] Brit. Med. Journ., July 10, 1897; and Centralb. f. Chir., No. 24, 1897. [13] Annals of Surg., July, 1897, p. 31. [14] Amer. Jour, Med. Sci., Sept., 1897.

### CEREBRAL SURGERY.

**Meningitis.**—In the discussion[1] which followed Carr's paper,[2] based on a series of cases of non-tuberculous posterior-basal meningitis, some interesting points were raised. Dr. Barlow summarised the symptoms which characterise the condition as (1) opisthotonos, which dominates the clinical picture and is a constant, early, and aggressive feature; (2) vomiting; and (3) the tendency to the occurrence of tonic spasm. This tonic spasm is different from the spasms, clonic in character, and followed by paresis, which occur in tuberculous meningitis. The limbs are rigidly extended. Anatomically, this condition is characterised by its being most marked in the posterior basal fossæ, and pathologically by the marked tendency to the formation of cicatricial adhesions, especially in the region of the roof of the fourth ventricle, where they cause blockage of foramina, and so tend to the production of hydrocephalus. The foramen of Monro, the aqueduct of Sylvius, and the foramen of Magendie have all been found blocked in this way. In other cases the floor and roof of the fourth ventricle have been fused; or the cervical spinal meninges so adherent as to interfere with free flow of the cerebro-spinal fluid. As regards the etiology, Barlow does not consider syphilis an important factor, nor is there always the history of a previous acute illness present, as in purulent meningitis, the disease often attacking apparently healthy children. Trauma may play a part; but he considers catarrh of the fauces or middle ear the most probable starting point; and this is a point to be borne in mind in considering the lines of treatment. In eight cases treated early by puncture of the tympanic membranes recovery took place. Lees and Ballance both confirm this opinion of the value of paracentesis of the tympanic membranes in treatment. Lees has observed that optic neuritis is very rare, and the nystagmus so often present he attributes to cerebellar disturbance. Blindness frequently ensues, even in the absence of optic neuritis. Abercrombie believes the disease is commoner in spring than at other seasons.

**Acute Purulent Meningitis.**—Berger[3] reports on the case of a woman, aged 33, from whose frontal sinus Luc removed a spindle-celled sarcoma. Suppuration followed, and on opening up the wound it was found that recurrence had taken place in the orbit. This was removed and drainage established, but meningitis and paresis on the left side ensued on the eighth day. The dura was opened and drained, but the recovery was delayed by an attack of infective pneumonia. The case illustrates the risks in operating on the frontal sinus, the disadvantages of attempting to get primary union, and the value of prompt and thorough operation should intra-cranial complications supervene.

**Sinus Thrombosis.**—Walter Downie,[4] of Glasgow, reports a case in which deep-seated mischief, beginning as a middle-ear catarrh, the result of influenza, ended in extensive septic thrombosis of the lateral sinus, which was successfully treated by operation. An unusual feature of the case was that the temperature remained persistently subnormal for two weeks after operation.

**Operations on the Mastoid.**—Lambert Lack[5] contributes a paper on the indications for and methods of operating on the mastoid. The indications for opening the antrum depend greatly on whether the suppuration in the middle ear is acute or chronic. In acute cases symptoms often subside spontaneously, in chronic cases rarely. He sums up in acute cases: (1) That immediate operation is necessary when the classical signs of acute mastoid abscess are present; or (2) when there are symptoms pointing to pyæmia, meningitis, &c.; (3) that local signs of mastoiditis with little general disturbance, or symptoms of retained pus without definite local signs, indicate the trial of simple measures before operation is performed; (4) operation is very rarely required, and almost only in those acute cases arising during the course of septic fevers; (5) that simple opening of the antrum is quite sufficient, and, as a rule, gives excellent results.

In cases of long-standing purulent otitis the position is very different. Operation is much more urgently demanded, and must be more thorough. The choice is between removal of the membrane and ossicles and throwing the antrum, middle ear, and external meatus into one cavity. The indications for removal of membrane and ossicles are (1) where ossicles are carious; (2) where there is a perforation in Shrapnell's membrane, as indicating suppuration in the attic; (3) where repeated slight attacks of earache, diminished secretion, increasing deafness, and headache are present; and (4) where cholesteatomatous masses fill the tympanum, or polypi recur after removal, or suppuration persists in spite of other treatment.

The more radical operation is indicated (1) when symptoms of acute mastoid abscess arise; (2) when aural polypi persistently recur; (3) when cholesteatomata involve the antrum; (4) in certain cases of atresia of the external auditory meatus; and (5) in all cases where chronic suppuration has resisted careful and prolonged treatment.

[1] Lancet, May 1, 1897. [2] Lancet, April 17, 1897. [3] Med. Week, March 5, 1897. [4] Glasgow Med. Jour., Sept., 1897. [5] Clinical Journal, Sept. 8, 1897.

